# Atlantic Dung Beetle Traits: A comprehensive dataset of functional traits for dung beetles (Coleoptera, Scarabeidae, Scarabaeinae) in the Atlantic Forest

**DOI:** 10.3897/BDJ.13.e170578

**Published:** 2025-11-10

**Authors:** Paula Ribeiro Anunciação, André Luiz Batista Tavares, Maria Eduarda Maldaner, Fernando Z. Vaz-de-Mello, Milton Cezar Ribeiro, Raffael Ernst

**Affiliations:** 1 Senckenberg - Leibniz Institution for Biodiversity and Earth System Research, Dresden, Germany Senckenberg - Leibniz Institution for Biodiversity and Earth System Research Dresden Germany; 2 Lancaster University, Lancaster, United Kingdom Lancaster University Lancaster United Kingdom; 3 Universidade Estadual Paulista (Unesp), Rio Claro, Brazil Universidade Estadual Paulista (Unesp) Rio Claro Brazil; 4 Universidade Federal de Mato Grosso, Cuiaba, Brazil Universidade Federal de Mato Grosso Cuiaba Brazil; 5 Faculty of Biology, Dresden University of Technology, Dresden, Germany Faculty of Biology, Dresden University of Technology Dresden Germany

**Keywords:** Tropical Forest, functional diversity, functional attributes, phylogenetic diversity, interspecific variation, intraspecific variation

## Abstract

**Background:**

Functional traits offer critical insights into species performance, survival strategies and ecological interactions. However, the availability of comprehensive trait datasets remains limited, primarily due to the substantial effort required for field sampling and laboratory analysis. This constraint is particularly pronounced in biodiversity hotspots such as the Atlantic Forest, one of the most diverse and threatened biomes globally. The **ATLANTIC DUNG BEETLE TRAITS** dataset addresses part of this gap by compiling extensive morphological and ecological information for a key insect group in this biome. Dung beetles (Coleoptera, Scarabaeinae) play essential roles in nutrient cycling, secondary seed dispersal and soil aeration, but many species are increasingly threatened by habitat loss, fragmentation and other human-induced pressures.

**New information:**

The dataset includes 47 distinct traits for 1,398 individuals representing up to 385 taxa. Many traits were measured separately by sex and/or habitat to capture intraspecific variation, resulting in 107 trait records overall. It is organised into two main components:

1. **Novel dataset** – Includes 371 individuals from 72 taxa, with measurements for six continuous morphological traits (mean biomass, wing length, wing width, wing area, wing aspect ratio and wing loading) and four categorical ecological traits (body size, diet, relocation behaviour and diel activity), all recorded at the individual level.

Additionally, this dataset includes a set of high-resolution photographs of dung beetle wings from 355 individuals across 51 taxa. These images were used to derive key morphological measurements and are provided to support reproducibility and further research.

1. **Published datasets** – Include records for 1,027 individuals representing up to 357 taxa, including 210 taxonomically uncertain species. These datasets cover both individual- and species-level functional traits, totalling 46 traits compiled from 29 published studies conducted between 2011 and 2024.

This functional trait database offers a standardised, detailed resource to support macroecological, functional and conservation analyses, contributing to biodiversity assessment and management in the Atlantic Forest.

## Introduction

Functional traits are species characteristics linked to performance and survival, such as morphological, physiological, phenological, behavioural and habitat attributes (Díaz and Cabido 2001, Violle et al. 2007). They are widely used to understand species–environment interactions and ecosystem functioning (Díaz and Cabido 2001, Violle et al. 2007). Although their formalisation gained prominence only in recent decades, the concept traces back to early naturalists like Darwin and Humboldt (Lavorel and Garnier 2002, McGill et al. 2006). Trait-based ecology advanced through Tilman’s work (Tilman et al. 1997) and was further strengthened by global databases such as TRY and the Open Traits Network (Kattge 2019, Opentraits.org 2024). Nevertheless, functional trait data remain scarce and often depend on labour-intensive measurements (McGill et al. 2006).

Dung beetles (Scarabaeidae, Scarabaeinae), with nearly 7,000 described species, illustrate this limitation, as information on their natural history and functional attributes remains incomplete (Pessôa et al. 2017, Schoolmeesters 2023). Despite their small size, they provide critical ecosystem services, including nutrient cycling, decomposition, soil aeration, seed dispersal, pest control and greenhouse gas mitigation (Nichols et al. 2008, Slade et al. 2016, Verdú et al. 2020). Their dependence on mammalian dung, susceptibility to microclimatic conditions and pesticides and vulnerability to habitat loss and fragmentation make them sensitive bioindicators (Carvalho et al. 2020). As such, dung beetles are valuable models for linking biodiversity to ecosystem functioning (Spector 2006).

The Brazilian Atlantic Forest, a global biodiversity hotspot, highlights the urgency of such data. Once widespread along Brazil’s eastern coast and extending into Paraguay and Argentina, less than 25% of its original cover remains (Marques and Grelle 2021, Vancine et al. 2024). This severe habitat loss threatens biodiversity and ecosystem processes. Functional trait data can, therefore, strengthen conservation by supporting evidence-based management, adaptive strategies and long-term biodiversity assessments in this biome (Arroyo-Rodríguez et al. 2017). Here, we present a comprehensive database of morphological and ecological traits for Atlantic Forest dung beetles, encompassing life histories, trophic strategies and behavioural adaptations to better understand their ecological roles and responses to environmental change.

## General description

### Purpose

**Figure 1.**
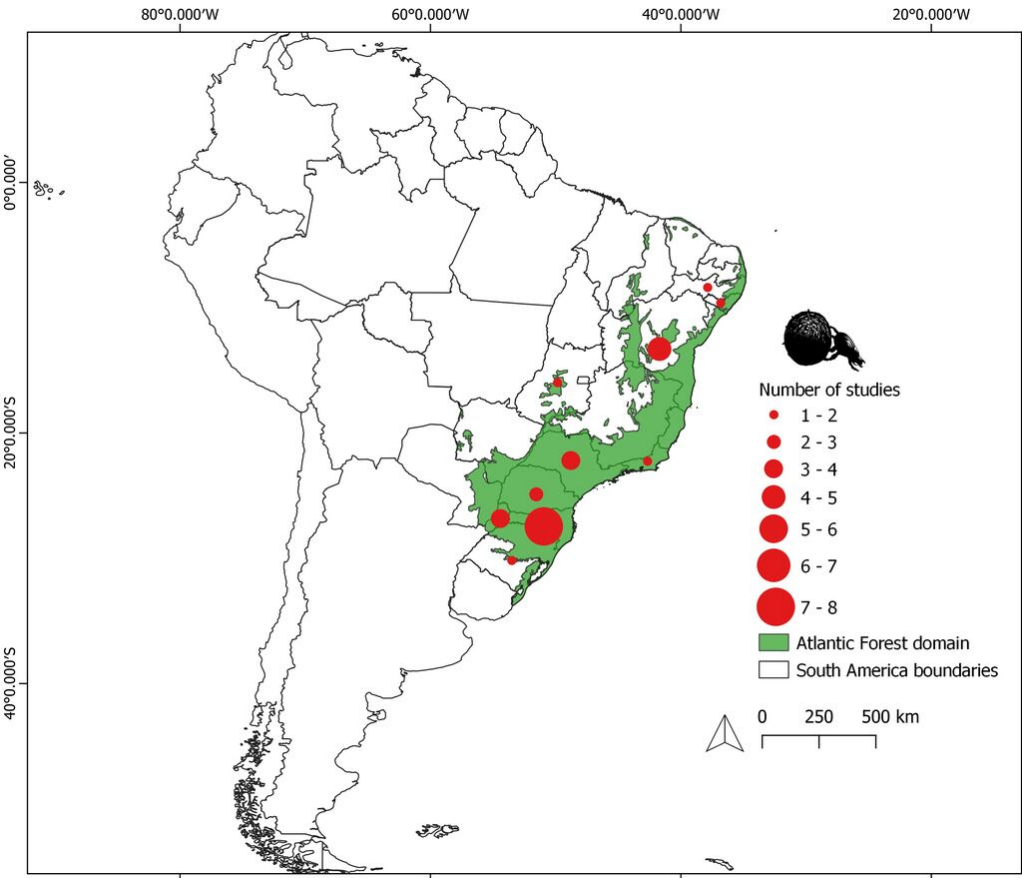
Distribution of the original Atlantic Forest domain in South America, with red dots indicating the sampling region locations from the ATLANTIC DUNG BEETLE TRAITS dataset.

This dataset compiles functional trait data of dung beetles (Scarabaeinae) from the Atlantic Forest biome (Fig. 1). It includes information from 29 peer-reviewed articles published between 2011 and 2024, as well as unpublished novel data collected by ALBT.

In total, the dataset contains measurements from 1,398 individuals representing up to 385 taxa and 47 functional traits. It is also supplemented by 371 individuals representing 72 taxa, derived from field collections conducted by ALBT. For this novel dataset, traits include four categorical variables (body size, diet, relocation behaviour and diel activity) and six continuous variables (mean biomass, wing length, wing width, wing area, wing aspect ratio and wing loading). Additionally, wing images and associated metadata for 353 individuals from 51 taxa were generated by MEM to facilitate the visualisation and measurement of wing-related traits, as well as to support future research.

### Additional information

Holding the majority of the distribution range of the Atlantic Forest, Brazil also accounts for most of the studies (n = 26). The federal states of Santa Catarina (n = 8), Bahia (n = 5) and São Paulo (n = 4) stand out for the number of studies conducted there. Apart from Brazil, Argentina presents a significant number of studies, particularly in the province of Misiones (n = 4) (Fig. 1).

## Sampling methods

### Sampling description

This dataset compiles functional trait data on dung beetles (Scarabaeinae) from eight Brazilian states and one Argentine province, all within the Atlantic Forest biome (Fig. 1). To assemble the dataset, we conducted a comprehensive literature search in Google Scholar using the keywords “functional traits”, “dung beetles”, and “Atlantic Forest”, combined with the Boolean operator “AND”. To improve coverage, we also searched in Portuguese and Spanish. Portuguese terms included: “besouro rola bosta” OR “Scarabaeinae”, AND “Mata Atlântica”, AND “traços funcionais” OR “características funcionais”; Spanish terms included: “escarabajos peloteros” OR “escarabajos estercoleros” OR “Scarabaeinae”, AND “Mata Atlántica” OR “Bosque Atlántico”, AND “rasgos funcionales”.

When a Master's or PhD thesis was later published in a peer-reviewed journal, we prioritised the published version to ensure the use of validated data. No temporal restrictions were applied and the final selection includes 29 peer-reviewed articles published between 2011 and 2024. We only included studies reporting functional traits of dung beetles within the Atlantic Forest.

The dataset includes 1,027 individuals from 137 valid species and 210 unresolved taxa at genus level and encompasses 46 functional traits. Apart the novel database, only two other studies have presented functional traits at the individual level: Giménez Gómez et al. (2020), with 17 species and 128 individuals and Silva and Hernández (2015), with eight species and 108 individuals. For a comprehensive understanding of traits and measurement methodologies in the scientific articles surveyed, please refer to the original publications and Traits coverage section.

The published data containing species-level traits are: Filgueiras et al. (2011), Hernández et al. (2011), Silva and Di Mare (2012), Korasaki et al. (2013), Campos and Hernández (2013), Silva et al. (2013), Audino et al. (2014), Lugon et al. (2017), Audino et al. (2017), Batilani-Filho and Hernandez (2017), Farias and Hernández (2017), Leite et al. (2018), Silva et al. (2018), Raine et al. (2018), Silva et al. (2019), Hernández et al. (2019), Soledad Soto et al. (2019), Raine et al. (2020), Gómez-Cifuentes et al. (2020), Souza et al. (2020), Sarmiento-Garcés and Hernández (2021), Alonso et al. (2022), Araújo et al. (2022), Filgueiras et al. (2022), Barreto et al. (2023), Pessôa et al. (2023), Maldaner et al. (2024).

All species records from the published datasets were carefully reviewed by FZVM and, when possible, identified to the species level. When an identification was updated, the original identification is retained in the column “ID at source”.

The novel dataset includes 371 individuals representing 63 valid species and 19 unresolved taxa, sampled across 30 sites in northeast São Paulo, Brazil, from February 2016 to January 2017. At each site, four pitfall traps (11 cm × 19 cm) spaced 50 m apart were buried, covered and filled with a killing solution; one trap used 50 g of human and pig faeces, the others using 500 g of cattle dung, left for 48 h to capture beetles active throughout the day (Marsh et al. 2013). The dataset provides four categorical traits (body size, diet, relocation behaviour, diel activity) and six continuous traits (mean biomass, wing length, wing width, wing area, wing aspect ratio, wing loading). Wing images of 353 individuals from 51 taxa were used to measure wing traits, mounted on slides for measurement and specimens kept as vouchers. Continuous traits were measured following standardised protocols using a Leica M205C stereomicroscope and MC190 HD camera, excluding wing setae. Up to 15 individuals per species were weighed (0.001 g precision) to calculate mean species biomass.

Specimens were identified to the genus level by FZVM using Costa-Silva et al. (2024) and to the species level using the literature cited therein (Vaz-De-Mello 2008, Génier 2009, Vaz-De-Mello et al. 2011), as well as more recent generic or species-group revisions (González-Alvarado and Vaz-De-Mello 2014, Silva et al. 2015, Rossini and Vaz-de-Mello 2015, Silva and Hernández 2015, Nunes and Vaz-De-Mello 2016, Valois et al. 2017, Vaz-De-Mello and Silva 2017, Maldaner et al. 2017, Cupello and Vaz-De-Mello 2018, González-Alvarado et al. 2019, Nunes and Vaz-de-Mello 2019, Pacheco and Vaz-de-Mello 2019, Vaz-De-Mello et al. 2020, González-Alvarado and Vaz-De-mello 2021, Arias-Buriticá and Vaz-de-Mello 2023, Cupello et al. 2023, Arias-Buriticá and Vaz-de-Mello 2024, Costa-Silva et al. 2024). Identification was further verified through comparisons with reference collections at CEMT (Coleção Entomológica de Mato Grosso Eurides Furtado, Universidade Federal de Mato Grosso, Cuiabá, Brazil) and a collection of type-specimen photographs. Most specimens are deposited at CEMT.

The final dataset contains trait data from 1,398 individuals, representing up to 385 taxa (including unresolved taxa at the genus level) and encompasses 53 functional traits.

## Geographic coverage

### Description

The dataset focuses on the Atlantic Forest, a biodiversity hotspot spanning 1.56 million km² across Brazil, northern Argentina and south-eastern Paraguay, which has lost over 75% of its original area and is highly fragmented (Vancine et al. 2024). The biome features diverse climates, altitudes from sea level to 2,900 m, multiple forest types and mean annual temperatures of 12.4–28.7°C with 1,000–4,200 mm of rainfall (Ribeiro et al. 2011, MapBiomas Project 2024). Major threats include deforestation, habitat fragmentation and human-driven land-use and climate changes, with most remaining fragments under 50 ha and limited connectivity, protected areas and indigenous territories covering only 10% of the biome (Marques and Grelle 2021, Vancine et al. 2024).

### Coordinates

33° S and 3° S Latitude; 58° W and 34° 45′ W Longitude.

## Taxonomic coverage

### Description

The dataset includes 1,398 individuals, corresponding to up to 385 taxa identified to genus level or higher. Overall, the species with the highest number of records in the dataset are Canthon (Francmonrosia) rutilans cyanescens Schmidt, 1922 (n = 38) and Dichotomius (Selenocopris) sericeus (Harold, 1867) (n = 38), followed by Deltochilum (Euhyboma) brasiliense (Castelnau, 1840) (n = 37), Deltochilum (Rubrohyboma) rubripenne (Gory, 1831) (n = 36), Deltochilum (Deltohyboma) morbillosum Burmeister, 1848 (n = 35) and Dichotomius (Dichotomius) mormon (Ljungh, 1799) (n = 34). The tribes Deltochilini and Coprini were the most represented, with 522 and 473 individuals, respectively.

### Taxa included

**Table:** 

Rank	Scientific Name	Common Name
subfamily	Scarabaeinae	Dung beetles

## Traits coverage

The dataset includes a wide range of morphological, ecological, behavioural and habitat-related traits. Below, traits are grouped into categories with definitions where relevant.

**Morphological traits** – traits describing external and internal organismal measurements or categorical structural features.

Abdomen slope (°C/°C): slope of abdominal temperature relative to ambient temperature, available per sex.

Anterior femur area (mean) (mm²): average area of the anterior femur, available per sex and per habitat.

Anterior tibia area (mean) (mm²): average area of the anterior tibia, available per sex and per habitat.

Anterior tibia length (mm): length of the anterior tibia, available per sex and per habitat.

Body area (mm²): projected body area, available per sex and per habitat.

Body size (mm): body size expressed in millimetres and categorised into classes, includes maximum, minimum and standard deviation, available per sex.

Body thickness (mm): body thickness, available per sex and per habitat.

Centroid size (mm): square root of the sum of squared distances of all landmarks from the centroid, available per sex.

Elytra width (mm): maximum width of the elytra, available per sex and per habitat.

Eye dorsal area (mm²): area of the compound eye measured dorsally, available per sex.

Head area (mm²): projected head area, available per sex and per habitat.

Head length (mm): linear distance from anterior to posterior head margin, available per sex.

Head width (mm): maximum width of the head, available per sex.

Metatibia length (mm): length of the metatibia, available per sex.

Pronotum width (mm): maximum width of the pronotum, available per sex and per habitat.

Prosternum height (mm): height of the prosternum, available per sex.

Protibia area (mm²): surface area of the protibia, available per sex.

Sphericity (mean, dimensionless): (S² / LI)¹ᐟ³, L = length, I = elytra width, S = thickness, available per sex and per habitat.

Thorax slope (°C/°C): thorax temperature slope.

Tooth width (mean) (mm): mean width of tooth structures, available per sex and per habitat.

Total length (mean) (mm): total body length, available per sex and per habitat.

Volume (mm³): calculated three-dimensional body volume, available per sex.

Wing area (mm²): surface area of the wings, available per sex.

Wing aspect ratio (dimensionless): (4 × wing length²) ÷ total wing area, available per sex, mean values also available per gram of body mass.

Wing length (mm): maximum linear extent of the wing, available per sex.

Wing loading (g/mm²): the ratio between body mass and total wing area, available per sex, mean values across individuals.

Wing shape (ratio, dimensionless): the ratio of wing length to wing width, available per sex.

Wing width (mm): maximum width of the wing, available per sex.

**Ecological traits** – traits describing the interaction of species with their environment, including resource use, habitat occupation and ecological strategies.

Biomass (g): biomass estimates.

Burial depth (cm): depth at which individuals bury dung or organic material.

Diet: classification of feeding guild.

Diel activity: temporal pattern of activity.

Endothermy (°C): the difference between the average temperature of the thorax and the nearby environment during flight.

Excavation capacity (ratio): inferred from the ratio of the anterior tibia to the length of the body.

Habitat specificity: degree of restriction to specific habitat types.

Levins' standardised index (dimensionless): measure of niche breadth, standardised from 0 (specialist) to 1 (generalist).

Nest: presence/absence of nests.

Pear ball nest: presence/absence of pear-shaped ball nests.

Primary disperser (%): percentage of the dung beetle species found in Brachyteles (muriqui) or tapir faeces.

Relative abundance (%): proportion of individuals of each species at a site, species with < 5% of total individuals being considered rare.

Spatial range distribution: classified as narrow (one region), intermediate (two adjoining regions) or large (three or more regions).

**Behavioural traits** – traits describing locomotion, movement, dispersal and other types of behaviour.

Ability to roll (ratio): estimated from the ratio of posterior tibia length to body length.

Direction (°): predominant movement direction.

Distance moved (m): distance travelled over 24 h between samplings.

Horizontal displacement (m): lateral displacement during locomotion.

Movement rate (m/day): rate of displacement over time.

Muscle strength (ratio): estimated from thorax size, calculated as (thorax height × elytra width) ÷ body length.

Relocation behaviour: food resource allocation behaviour.

Up down: classification of vertical movement (upward vs. downward displacement).

Flight capacity (ratio): estimated as the ratio of wing size to body size.

Minimum thoracic take-off temperature (°C): temperature at which a tethered beetle can initiate flight.

**Taxonomic traits**

Sex: male or female.

Tribe: taxonomic tribe of the species.

Age: age class of individuals.

### Trait observation summary

**Figure 2.**
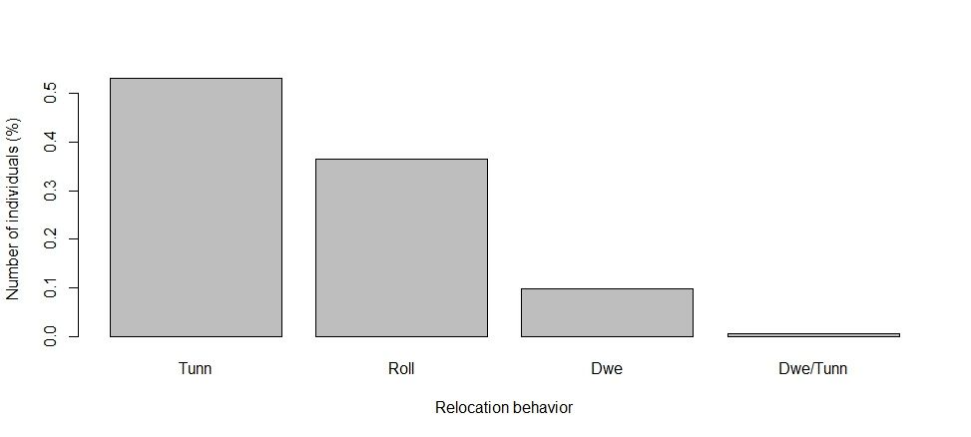
Number of individuals in each category of relocation behaviour for dung beetles in the Atlantic Forest. Categories are defined as follows: Tunn = Tunnellers, Roll = Rollers, Dwe = Dwellers.

**Figure 3.**
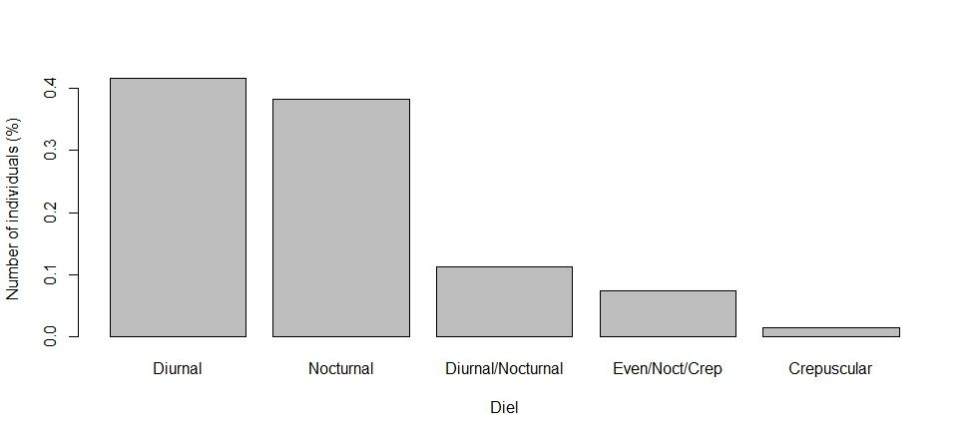
Number of individuals in each category of period of activity for dung beetles in the Atlantic Forest. Categories are defined as follows: Even/Noct/Crep = Evening/Nocturnal/Crepuscular.

**Figure 4.**
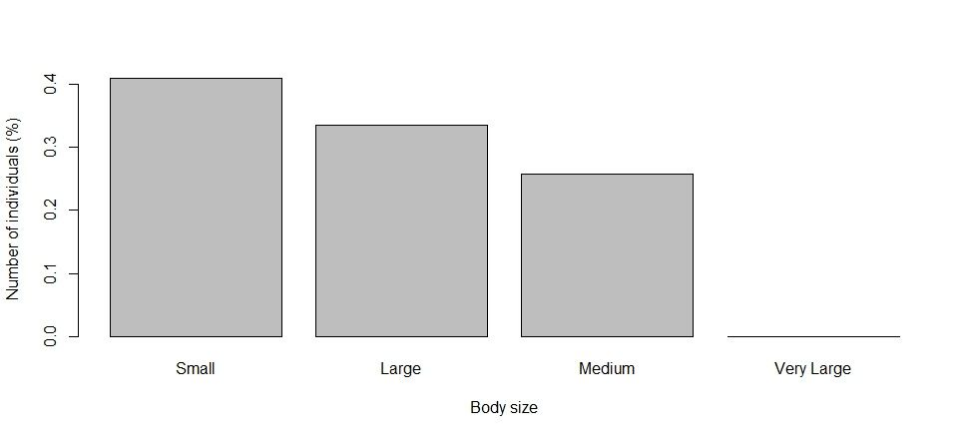
Number of individuals in each category of body size for dung beetles in the Atlantic Forest.

**Figure 5.**
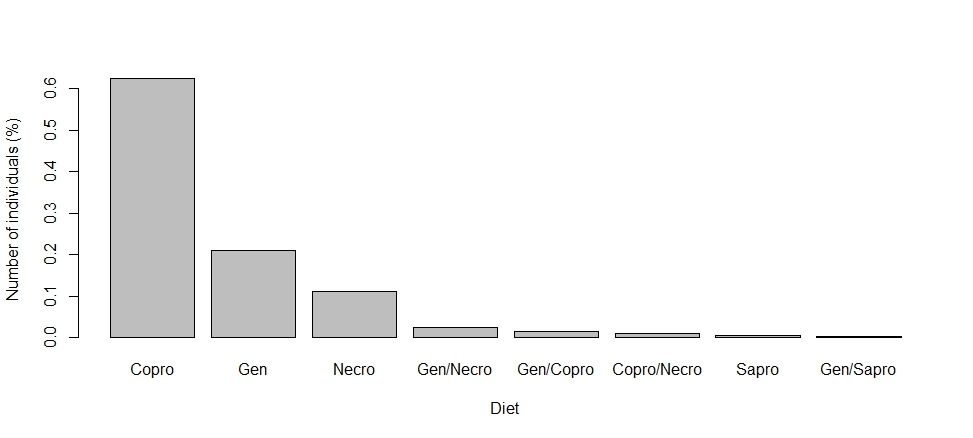
Number of individuals in each category of diet for dung beetles in the Atlantic Forest. Categories are defined as follows: Copro = coprophagous, Gen = generalist, Necro = necrophagous, Sapro = saprophagous.

**Figure 6.**
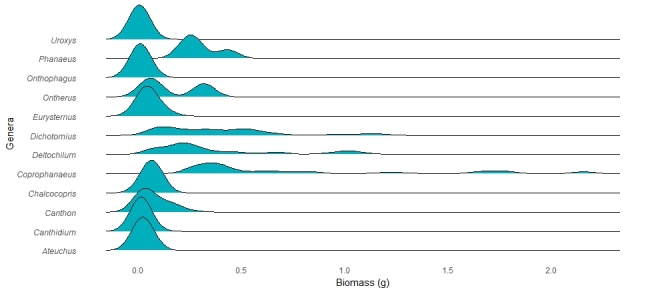
Variation in body mass (g) amongst the most abundant dung beetle genera documented in the ATLANTIC DUNG BEETLE TRAITS database.

The functional traits with the highest number of observations are predominantly categorical. Leading the list is relocation behavior (n = 1041), with most dung beetles identified as tunnellers (n = 560, Fig. 2). The second most reported trait is the period of activity (n = 805), primarily diurnal species (n = 335, Fig. 3). Next is categorical body size (n = 694), with the majority being small (n = 277, Fig. 4). The diet trait is also frequently documented (n = 671), with most beetles being coprophagic (n = 419, Fig. 5). Amongst continuous traits, mean biomass is the most reported (n = 369, mean = 0.109 g, Fig. 6).

## Usage licence

### Usage licence

Creative Commons Public Domain Waiver (CC-Zero)

## Data resources

### Data package title

Atlantic Dung Beetle Traits

### Resource link

https://figshare.com/s/61c46715e86604f1ad6d

### Number of data sets

3

### Data set 1.

#### Data set name

Comprehensive Functional Trait Dataset of Atlantic Forest Dung Beetles

#### Data format

txtfile

#### Download URL

https://figshare.com/s/61c46715e86604f1ad6d

#### Description

The dataset provides morphological, ecological, behavioural and taxonomic information for dung beetle species from the Brazilian Atlantic Forest, including measurements, biomass, ecological roles, movement traits and metadata such as species, sex, habitat and collection locality.

### Data set 2.

#### Data set name

Wing Image Collection of Neotropical Dung Beetles

#### Data format

MS

#### Download URL

https://figshare.com/s/01a7064dcec5f7bcba58

#### Description

The dataset contains Word files with high-resolution images of dung beetle wings, along with metadata including a description field with collection locality.

### Data set 3.

#### Data set name

Atlantic Dung Beetle traits - data dictionary

#### Data format

PDF

#### Download URL

https://figshare.com/s/a98f5a6cd4e676edf90c

#### Description

Detailed descriptions of all fields included in the Atlantic Dung Beetle Trait dataset.
